# Biomarker discovery and development for frontotemporal dementia and amyotrophic lateral sclerosis

**DOI:** 10.1093/brain/awac077

**Published:** 2022-02-24

**Authors:** Jared S. Katzeff, Fiona Bright, Katherine Phan, Jillian J. Kril, Lars M. Ittner, Michael Kassiou, John R. Hodges, Olivier Piguet, Matthew C. Kiernan, Glenda M. Halliday, Woojin Scott Kim

**Affiliations:** 1 Brain and Mind Centre, The University of Sydney, Sydney, NSW 2050, Australia; 2 School of Medical Sciences, The University of Sydney, Sydney, NSW 2006, Australia; 3 Dementia Research Centre, Macquarie Medical School, Macquarie University, Sydney, NSW 2109, Australia; 4 School of Chemistry, The University of Sydney, Sydney, NSW 2006, Australia; 5 School of Psychology, The University of Sydney, Sydney, NSW 2006, Australia; 6 Institute of Clinical Neurosciences, Royal Prince Alfred Hospital, Sydney, NSW 2050, Australia

**Keywords:** frontotemporal dementia, amyotrophic lateral sclerosis, biomarkers, neurofilament, proteomics

## Abstract

Frontotemporal dementia refers to a group of neurodegenerative disorders characterized by behaviour and language alterations and focal brain atrophy. Amyotrophic lateral sclerosis is a rapidly progressing neurodegenerative disease characterized by loss of motor neurons resulting in muscle wasting and paralysis. Frontotemporal dementia and amyotrophic lateral sclerosis are considered to exist on a disease spectrum given substantial overlap of genetic and molecular signatures. The predominant genetic abnormality in both frontotemporal dementia and amyotrophic lateral sclerosis is an expanded hexanucleotide repeat sequence in the *C9orf72* gene. In terms of brain pathology, abnormal aggregates of TAR-DNA-binding protein-43 are predominantly present in frontotemporal dementia and amyotrophic lateral sclerosis patients. Currently, sensitive and specific diagnostic and disease surveillance biomarkers are lacking for both diseases. This has impeded the capacity to monitor disease progression during life and the development of targeted drug therapies for the two diseases. The purpose of this review is to examine the status of current biofluid biomarker discovery and development in frontotemporal dementia and amyotrophic lateral sclerosis. The major pathogenic proteins implicated in different frontotemporal dementia and amyotrophic lateral sclerosis molecular subtypes and proteins associated with neurodegeneration and the immune system will be discussed. Furthermore, the use of mass spectrometry-based proteomics as an emerging tool to identify new biomarkers in frontotemporal dementia and amyotrophic lateral sclerosis will be summarized.

## Teaser

Frontotemporal dementia and amyotrophic lateral sclerosis are neurodegenerative diseases that lie on the same disease spectrum. Katzeff, Bright *et al.* explore and evaluate potential biomarkers that could distinguish the two diseases and ultimately improve patient diagnosis and treatment.

## Introduction

Frontotemporal dementia refers to a group of neurodegenerative disorders characterized by altered behaviour and language, with a progressive decline in executive function.^1^ Frontotemporal dementia is the second most common form of younger-onset dementia after Alzheimer’s disease, frequently occurring before 65 years of age.^2^ Frontotemporal dementia is categorized clinically into various subtypes; the main three include behavioural-variant frontotemporal dementia and two language variants, semantic dementia (also known as semantic variant primary progressive aphasia) and progressive non-fluent aphasia (also known as non-fluent variant primary progressive aphasia). In addition, frontotemporal dementia overlaps with movement disorders having two additional subtypes, progressive supranuclear palsy and corticobasal degeneration. Behavioural-variant frontotemporal dementia is the most common form of frontotemporal dementia presenting with a range of symptoms that include disinhibited behaviour, apathy, increased consumption of sweet foods and alcohol, loss of empathy and emotional processing, and impaired executive function.^1,3–5^ Semantic dementia is characterized by a loss of semantic knowledge that typically presents as progressive anomia, in the context of fluent expressive speech. In contrast, progressive non-fluent aphasia is characterized by effortful and distorted speech with or without agrammatism in the context of preserved comprehension.^4,6,7^

Amyotrophic lateral sclerosis, also known as motor neuron disease, is a rapidly progressing neurodegenerative disease characterized by degeneration of motor neurons in the brain and spinal cord, leading to muscle atrophy and paralysis.^8,9^ Fatality in amyotrophic lateral sclerosis frequently occurs within 3–5 years of diagnosis.^10,11^ Amyotrophic lateral sclerosis most commonly occurs around 60 years of age, affecting more males than females.^10,11^ Amyotrophic lateral sclerosis is categorized depending on whether symptoms are predominantly related to upper motor neurons or lower motor neurons. In ∼70% of amyotrophic lateral sclerosis cases, both upper motor neurons and lower motor neurons are affected, while minor subtypes predominantly involve either upper motor neurons or lower motor neurons.^9,12,13^ Amyotrophic lateral sclerosis often presents with either onset of limb weakness or bulbar symptoms affecting speech or swallowing.

Approximately 15% of frontotemporal dementia cases exhibit motor symptoms and 15–18% of amyotrophic lateral sclerosis cases exhibit frontotemporal dementia symptoms.^14^ Furthermore, 50% of amyotrophic lateral sclerosis cases have evidence of frontotemporal dementia-like cognitive changes.^11^ Pathologically, frontotemporal dementia cases are characterized by abnormal aggregates of TDP-43, tau or FET proteins (FET proteins refer to a group of three proteins: fused in sarcoma, EWSR1 and TAF15) while amyotrophic lateral sclerosis cases are pathologically characterized by TDP-43, superoxide dismutase-1 (SOD1) or FET proteins.^14–16^ Of particular interest, abnormal aggregates of TDP-43 are identified in ∼50% of frontotemporal dementia cases and 95% of amyotrophic lateral sclerosis cases.^17^ In 2011, TDP-43 aggregates were shown to be associated with the *C9orf72* repeat expansion in both frontotemporal dementia and amyotrophic lateral sclerosis.^18,19^ Together, these studies provide clinical, molecular and genetic evidence supporting the existence of frontotemporal dementia and amyotrophic lateral sclerosis on a disease spectrum. While genetic abnormalities in patients are useful in identifying the underlying molecular pathologies, biomarker development in association with genetic status and clinical assessment is necessary to identify and distinguish molecular signatures in patients who exhibit similar clinical symptoms. Sensitive and specific biomarkers have the potential to assist with targeting specific molecular subtypes for mechanistic treatments, tracking disease progression during life and streamlining patients for clinical trials that are currently lacking for both frontotemporal dementia and amyotrophic lateral sclerosis.

## Gene-based biomarkers

**Figure 1:**
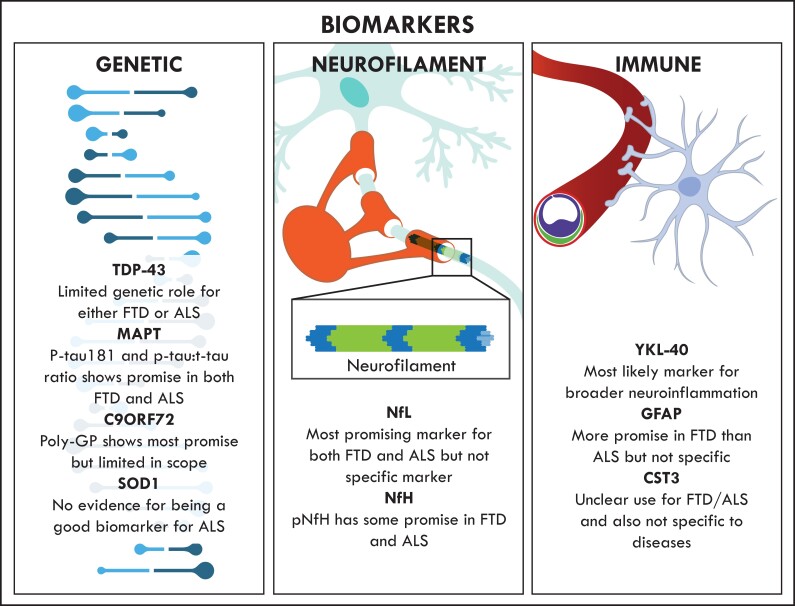
**An overview of current biomarker development in frontotemporal dementia and amyotrophic lateral sclerosis.** The main genetic biomarkers evaluated are TDP-43, MAPT, C9ORF72 and SOD1. Neurofilament biomarkers evaluated are NfL and NfH. The main immune-related biomarkers evaluated are YKL-40, GFAP and CST3. All of these markers have limitations in the diagnosis of frontotemporal dementia and amyotrophic lateral sclerosis, and therefore there is an urgent need for new biomarker development for the two diseases.

In general, gene-based biomarkers (Fig. 1) have the potential to determine disease susceptibility and assist with discriminating early-stage versus late-stage disease. Genetic mutations in *TARDBP*, *C9orf72*, *MAPT* and *SOD1* have been used to classify frontotemporal dementia and amyotrophic lateral sclerosis. While the genetic subtypes of frontotemporal dementia and amyotrophic lateral sclerosis continue to be extensively explored and advances have been made in correlating pathological subtypes with causal genes, the use of gene-based biomarkers, particularly those genes that overlap between frontotemporal dementia and amyotrophic lateral sclerosis, remains to be fully investigated. Although gene-based biomarkers provide promise for biomarker discovery in frontotemporal dementia and amyotrophic lateral sclerosis, it is likely that they will need to be used in combination with other types of biomarker that target additional pathways and mechanisms of disease.

### TARDBP

The *TARDBP* gene, which encodes TDP-43, accounts for ∼4% of familial amyotrophic lateral sclerosis, 1.5% of sporadic amyotrophic lateral sclerosis patients and ∼2% of behavioural-variant frontotemporal dementia patients.^20,21^ Pathologically, TDP-43 pathology is present in ∼50% of frontotemporal dementia cases and 95% of amyotrophic lateral sclerosis.^17^ TDP-43 is an RNA/DNA-binding protein that is widely expressed, particularly in the CNS. While the complete function of TDP-43 is yet to be determined, under physiological conditions TDP-43 is involved in RNA biogenesis and processing.^22,23^

Use of TDP-43 as a biomarker has been explored in serum, plasma and CSF, predominantly in frontotemporal dementia cases. In behavioural-variant frontotemporal dementia, serum and plasma levels of phosphorylated TDP-43 (pTDP-43) have been used to differentiate between genetic subtypes. While no significant difference pTDP-43 was observed between *C9orf72* and *GRN*-mutation carriers, both groups had higher levels of pTDP-43 compared to other behavioural-variant frontotemporal dementia subtypes and controls in both CSF and plasma samples. Additionally, pTDP-43 levels in plasma correlated positively with CSF; however, there was no correlation in total TDP-43 (tTDP-43) levels within the same samples.^24^ CSF tTDP-43 failed to differentiate between *C9orf72* expansion carriers and non-carriers. CSF tTDP-43 has also been shown to be elevated in amyotrophic lateral sclerosis compared to behavioural-variant frontotemporal dementia patients.^25^ CSF tTDP-43 was elevated in sporadic amyotrophic lateral sclerosis compared to age-matched controls and other neurodegenerative and inflammatory diseases, including Parkinson’s disease, multiple sclerosis and Guillain–Barré syndrome.^26,27^

There are, however, two major concerns with the use of TDP-43 as a biomarker. First, there is no consensus as to whether full length TDP-43, pTDP-43 or truncated variants of TDP-43 would be most effective for measurement.^28^ Second, directly measuring tTDP-43 in a biofluid carries the risk of measuring peripheral rather than brain derived TDP-43. As such, development of methods that can differentiate brain derived TDP-43 from other tissue sources are required to effectively use TDP-43 as a biomarker in behavioural-variant frontotemporal dementia and amyotrophic lateral sclerosis.^28,29^

### MAPT

The tau protein stabilizes microtubules,^30,31^ and over 50 *MAPT* mutations have been associated with frontotemporal dementia.^32–34^ Abnormally phosphorylated tau aggregates, which are the second most common form of pathological protein aggregate in frontotemporal dementia, are mostly associated with behavioural-variant frontotemporal dementia and progressive non-fluent aphasia subtypes.^16^ The ratio between phosphorylated tau (p-tau181) and total tau (t-tau) has been suggested for differentiation between molecular frontotemporal dementia subtypes. Frontotemporal dementia cases with TDP-43 pathological inclusions have a reduction in the ratio of p-tau181 and t-tau compared to cases with tau pathology.^35,36^ Additionally, plasma p-tau181 levels are only significantly altered in the *MAPT* genetic subtype of frontotemporal dementia.^37^ Tau can also aid in the differentiation between frontotemporal dementia and Alzheimer’s disease, with a higher t-tau:amyloid-β_1-42_ ratio is indicative of Alzheimer’s disease^38^ and plasma p-tau181 is elevated in Alzheimer’s disease compared to frontotemporal dementia and controls.^39,40^ In amyotrophic lateral sclerosis, there is also a significant reduction of both p-tau181 and the p-tau:t-tau ratio compared to frontotemporal dementia with 4-repeat tau inclusions and controls.^41^ However, the multiple fragments of tau, p-tau, t-tau and non-phosphorylated tau alone are, so far, unable to effectively discriminate between frontotemporal dementia and Alzheimer’s disease, suggesting that tau alone is unlikely to be a disease specific biomarker.^42^

### C9ORF72

The *C9orf72* gene consists of 12 exons, of which exons 1a and 1b are alternatively spliced, producing three transcripts and two isoforms.^18,43^ A G_4_C_2_ hexanucleotide repeat expansion is located between exons 1a and 1b, and there are usually 2–20 repeats in healthy individuals. However, in behavioural-variant frontotemporal dementia and amyotrophic lateral sclerosis, hundreds or even thousands of repeats are present; the exact minimum number required for pathology is currently undefined.^7^ The physiological role of C9ORF72 protein is unclear; however, it may modulate neuronal morphogenesis.^44^ In addition, *C9orf72* has a potential role in neuroinflammation, as demonstrated by *C9orf72* knockout rodent studies that exhibit a systemic proinflammatory state, severe autoimmune disease in some strains^45,46^ and mild neuroinflammation with increased expression of key inflammatory mediators in microglia, and an upregulation of inflammatory genes compared to control cohorts.^47^ Furthermore, *C9orf72* has been identified to be required for normal function of myeloid cells, and altered microglial function is suggested to contribute to neurodegeneration in *C9orf72* expansion carriers.^47^ Therefore, C9ORF72 may have the potential to also be used as a marker of altered immune status in frontotemporal dementia and amyotrophic lateral sclerosis; however, this remains to be investigated. Another aspect of *C9orf72* that could contribute towards the development biomarkers for frontotemporal dementia and amyotrophic lateral sclerosis is the study of methylation of G_4_C_2_ hexanucleotide repeat. Methylation studies have revealed that large repeats are methylated whereas small or intermediate repeats are not, providing a new avenue for distinguishing repeat sizes.^48,49^ The same phenomenon was observed in blood, brain and spinal cord tissues of an individual.^49^ Further investigation into *C9ORF72* methylation may help to establish a more accurate cut-off for pathogenic repeat.

Pathologically, *C9orf72* is the most common gene implicated in behavioural-variant frontotemporal dementia and amyotrophic lateral sclerosis, affecting ∼40% of familial amyotrophic lateral sclerosis and 25% of familial behavioural-variant frontotemporal dementia.^50^ A pathological mechanism of *C9orf72* gene expansion entails the translation of the expansion into dipeptide repeat proteins.^51–53^ Formation of dipeptide repeat protein occurs when repeat-associated non-ATG (RAN) translation of the hexanucleotide repeat forms five dipeptide repeat proteins: glycine-alanine (GA), glycine-arginine (GR), proline-alanine (PA), proline-arginine (PR) and glycine-proline (GP).^53^ Production of poly-PR and poly-GR leads to neurotoxicity via impaired protein translation.^54^ Poly-GP has attracted attention as a potential biomarker in *C9orf72* gene expansion carriers in both behavioural-variant frontotemporal dementia and amyotrophic lateral sclerosis. While asymptomatic mutation carriers have elevated CSF and peripheral blood mononuclear cell poly-GP, these levels were raised further in disease groups.^55–57^

### SOD1

SOD1 is an antioxidant that converts superoxide into molecular oxygen and hydrogen peroxide.^58^ There are numerous amyotrophic lateral sclerosis-linked *SOD1* mutations.^59,60^ SOD1 protein aggregates are characteristic of amyotrophic lateral sclerosis patients with *SOD1* mutations and are the second most common pathological subtype of amyotrophic lateral sclerosis. However, there are no significant changes in CSF SOD1 levels in amyotrophic lateral sclerosis, including sporadic or *SOD1* mutation carriers,^61^ suggesting that SOD1 may not be an effective biomarker for amyotrophic lateral sclerosis. Contrary to this, CSF SOD1 has previously been identified as a robust pharmacodynamic marker in response to antisense oligonucleotide treatment.^62^ CSF SOD1 has subsequently been used as a pharmacodynamic biomarker in a phase 1–2 ascending dose clinical trial of the SOD1 antisense oligonucleotide Tofersen. In the highest-dosage group, CSF SOD1 levels declined 36% with some evidence of a reduction in disease measures; however, a correlation between treatment and clinical outcomes could not be drawn due to the small study size.^63^ Therefore, the use of SOD1 as a biomarker may lie primarily in determining the pharmacodynamics of SOD1-lowering therapies as opposed to differentiating between subtypes of amyotrophic lateral sclerosis.

## Neurofilament-related biomarkers

Neurofilaments, consisting of neurofilament heavy, medium and light chains (NfH, NfM and NfL; Fig. 1) and α-internexin, are abundantly expressed in neurons and thought to function in axonal growth and maintenance.^64^ Increased neurofilament levels in CSF or blood have been used as a marker for neurodegeneration so far.^65^

### Neurofilament light chain

NfL has attracted considerable focus as a biomarker of neurodegeneration. NfL has been repeatedly shown to have important prognostic value in frontotemporal dementia. Serum NfL is inversely correlated with survival time.^66^ Furthermore, presymptomatic cases have shown elevated NfL within serum before disease manifestation.^67^ Consistent with this, serum NfL was one of the earliest identified altered markers in frontotemporal dementia GRN-mutation carriers.^68^ Plasma NfL also has prognostic value with plasma NfL elevation occurring in asymptomatic frontotemporal dementia mutation carriers preceding disease onset.^69^

NfL levels in serum correlate to those in CSF. Importantly, serum and CSF NfL levels correlate with disease severity, functional impairment and brain atrophy.^70,71^ For diagnosis, CSF NfL combined with p-tau181:t-tau ratio provided 80% sensitivity and 81% specificity in differentiating between frontotemporal dementia cases with tau or TDP-43 pathological inclusions, with NfL levels most significantly elevated in those with TDP-43 pathological inclusions.^72^ This is consistent with *C9orf72* gene expansion carriers found to display higher serum NfL than frontotemporal dementia patients without the mutation.

In amyotrophic lateral sclerosis, NfL has been predominantly studied in CSF and NfL is currently considered the most effective amyotrophic lateral sclerosis biomarker for diagnosis and predicting survival time.^73^ Elevated CSF NfL is indicative of amyotrophic lateral sclerosis severity and progression.^74^ Amongst neurodegenerative diseases, frontotemporal dementia and amyotrophic lateral sclerosis exhibit the greatest elevations in CSF NfL.^75^ Compared to healthy controls, NfL levels are ∼20-fold higher in amyotrophic lateral sclerosis CSF, whereas they are 3-fold higher in frontotemporal dementia CSF.^76^ In a Swedish cohort study, amyotrophic lateral sclerosis patients exhibited a 709% increase in CSF NfL compared to controls, whereas frontotemporal dementia patients had a 307% increase. In both diseases, elevated NfL inversely correlated to survival time, suggesting that NfL may be a relevant prognostic biomarker.^77^ NfL has also been studied in amyotrophic lateral sclerosis serum, correlating to disease progression and decreased survival time, but not disease severity.^78^ Blood and CSF NfL is amongst the earliest markers to change in patients transitioning from presymptomatic to symptomatic.^79^ Furthermore, NfL could be used to differentiate amyotrophic lateral sclerosis subtypes. For example, plasma NfL levels were significantly higher in bulbar-onset amyotrophic lateral sclerosis patients compared to spinal-onset amyotrophic lateral sclerosis patients.^80^ Also, NfL could potentially be used as a diagnostic biomarker to differentiate amyotrophic lateral sclerosis from clinically relevant amyotrophic lateral sclerosis mimics.^80^

However, it is important to note that an elevation in NfL levels is common in other neurodegenerative diseases, including Alzheimer’s disease,^81^ Parkinson’s disease^82^ and multiple sclerosis,^83^ suggesting that NfL is a general marker of neurodegeneration induced axonal damage rather than specific to disease processes in frontotemporal dementia or amyotrophic lateral sclerosis. Nevertheless, NfL is useful as a differentiating biomarker as elevations are greater in frontotemporal dementia and amyotrophic lateral sclerosis compared to atypical parkinsonism and various dementias especially when aged-related concentration cut-offs are considered.^84^ Overall, NfL is so far the most established biomarker in frontotemporal dementia and amyotrophic lateral sclerosis, although its utility across the spectrum of neurodegenerative diseases indicates that more specific biomarkers are required.

### Neurofilament heavy chain

As the NfH chain is phosphorylated, most studies have targeted phosphorylated NfH (pNfH). CSF pNfH has been shown to be elevated in frontotemporal dementia compared to early-onset Alzheimer’s disease.^85^ In amyotrophic lateral sclerosis, pNfH was elevated in serum, plasma and CSF. In this study, pNfH levels in all biofluids positively correlated with increases in disease progression.^86^ Both NfL and pNfH are also elevated in amyotrophic lateral sclerosis CSF and serum before symptom onset from nine months to 3.5 years.^87^ Receiver operating characteristics showed that CSF pNfH could differentiate amyotrophic lateral from amyotrophic lateral mimics.^80^ While further research is required, pNfH exhibits promise as a biomarker for both frontotemporal dementia and amyotrophic lateral sclerosis.

### Neurofilament medium chain

NfM is the least explored neurofilament biomarker so far. A recent study, based on antibody-suspension bead arrays, demonstrated that NfM is elevated in frontotemporal dementia CSF.^88^ Furthermore, in an earlier study, high levels of NfM were observed in amyotrophic lateral sclerosis plasma.^89^ Further research is required to establish any potential utility of NfM as a biomarker for frontotemporal dementia and amyotrophic lateral sclerosis.

## Immune-related biomarkers

Neuroinflammation is considered a pathological hallmark of neurodegenerative diseases and there has been increasing attention on the potential role of neuroinflammatory and peripheral inflammatory pathways in the pathogenesis of both frontotemporal dementia and amyotrophic lateral sclerosis, as reviewed elsewhere.^90,91^ As such, various immune-related biomarkers have been explored in both frontotemporal dementia and amyotrophic lateral sclerosis (Fig. 1); however, concerns remain as to the sensitivity and in particular specificity of such markers given their general levels across the spectrum of neurodegenerative diseases.

### YKL-40

Chitinase-3-like protein (YKL-40) is a glycoprotein thought to be involved in extracellular matrix remodelling and inflammation.^92–94^ YKL-40 CSF levels are increased in monogenic and sporadic cases of frontotemporal dementia and amyotrophic lateral sclerosis compared to both asymptomatic mutation carriers and controls.^95^ These findings have been pathologically validated with elevated YKL-40 prevalent in the amyotrophic lateral sclerosis motor cortex, frontal cortex and spinal cord.^96,97^ YKL-40 CSF levels are increased in frontotemporal dementia with TDP inclusions compared to controls. Additionally, when combined with p-tau and the p-tau/t-tau ratio, differentiated frontotemporal dementia from non-frontotemporal dementia dementias, including Alzheimer’s disease and dementia with Lewy bodies, with 90% sensitivity and 78% specificity.^98^ Interestingly, CSF YKL-40 levels are greater in frontotemporal dementia with *GRN* and *MAPT* mutations than in *C9orf72* expansion carriers.^99^ However, a recurring finding is that alterations in CSF are not reflected in plasma or serum.^95–97,100^ Furthermore, YKL-40 is also altered in Alzheimer’s disease, thus YKL-40 alone should be considered a marker of neurodegeneration relating to neuroinflammatory mechanisms rather than a disease specific biomarker.^101^

### GFAP

Glial fibrillary acidic protein (GFAP) is an intermediate filament protein released by astrocytes during astrogliosis.^102^ GFAP has been considered a potential frontotemporal dementia biomarker with elevated GFAP levels in the CSF^103^ and plasma of frontotemporal dementia *GRN-*mutation carriers.^104^ In serum, GFAP is correlated with cognitive state.^105^ In amyotrophic lateral sclerosis, GFAP is also elevated in CSF samples.^106^ However, since GFAP is a measure of astrogliosis, which is common in other neurodegenerative diseases including Alzheimer’s disease and dementia with Lewy bodies, it cannot be used as a specific biomarker for frontotemporal dementia or amyotrophic lateral sclerosis.^103^

### CST3

Cystatin C or cystatin 3 (CST3) is a cysteine protease inhibitor that is abundant in the CSF and implicated in cell signalling, inflammation and neuronal cell death.^107–110^ CST3 is thought to have a pathological role within the amyotrophic lateral sclerosis brain, being one of only two known proteins in Bunina bodies, an intraneuronal inclusion found only in amyotrophic lateral sclerosis.^111,112^ However, it is unclear whether CST3 is altered in CSF or serum in amyotrophic lateral sclerosis with different studies reporting contradictory results.^113–115^ Analysis of CST3 levels in frontotemporal dementia has not been well characterized to date. In one study, CST3 was shown to be decreased in frontotemporal dementia with *GRN* mutation compared to *C9orf72* repeat expansion carriers, suggesting that it may differentiate frontotemporal dementia subtypes.^116^ However, CST3 levels were also significantly lower in both CSF and serum in Alzheimer’s disease and dementia with Lewy bodies.^117^ Further research is needed to establish the use of CST3 as a biomarker for amyotrophic lateral sclerosis or frontotemporal dementia.

## Emerging biomarkers

An emerging biomarker for amyotrophic lateral sclerosis is T regulatory cells (Tregs). A number of studies has shown a significant and progressive reduction in number of Tregs, and that Tregs are less effective in promoting immune suppression in amyotrophic lateral sclerosis patients.^118–121^ Tregs levels have been shown to correlate with rate of disease progression and patient survival and are therefore considered a promising therapeutic target for neuroprotection in amyotrophic lateral sclerosis as reviewed in detail elsewhere.^122^ Aside from presenting as a promising therapeutic target, Tregs measurement in blood is also considered an important pharmacodynamic target of biomarkers across different clinical trials as recently reviewed,^123^ therefore having the potential to be informative in disease phenotype and clinical stratification.

Another emerging biomarker for amyotrophic lateral sclerosis is the urinary neurotrophin receptor p75 extracellular domain (p75^ECD^). There is evidence indicating a significant elevation in p75^ECD^ concentrations in the of amyotrophic lateral sclerosis patients, suggesting urinary p75^ECD^ concentration could be a potential biomarker for amyotrophic lateral sclerosis.^124,125^ Urinary p75^ECD^ concentrations reflected the disease severity and provided additional evidence for an amyotrophic lateral sclerosis diagnosis in patients with clinically suspected amyotrophic lateral sclerosis.^125^ In a recent meta-analysis, consisting of five case-control studies, urinary p75^ECD^ levels were shown to be significantly higher in patients with amyotrophic lateral sclerosis compared to non-neurological controls.^126^ The strong association between p75^ECD^ levels and amyotrophic lateral sclerosis supports further investigation of p75^ECD^ as a potential biomarker for amyotrophic lateral sclerosis as a diagnostic biomarker and a progression indicator.^126^ The practicalities of urinary biomarkers are advantageous given their non-invasive nature and ease of accessibility.

A biomarker strategy that is emerging in the field of neurodegenerative diseases is the polygenic risk score (PRS). PRS is based on a computational algorithm that combines vast measures of genome-wide genetic data to predict an individual’s inherited susceptibility to a disease. It has been widely applied to the analysis of cancer and cardiovascular disease and to a limited degree Alzheimer’s disease.^127^ It is beginning to be applied to frontotemporal dementia and amyotrophic lateral sclerosis. PRS is particularly useful in the study of these complex diseases, in which the genetic aetiology is multifactorial and heterogeneous. In one study, polygenic risk for frontotemporal dementia was shown to be associated with executive functioning, whereas polygenic risk for amyotrophic lateral sclerosis was associated with verbal-numeric reasoning.^128^ Future studies that combine PRS with pathway analysis will enable determining more enhanced therapeutic and preventative measures for frontotemporal dementia and amyotrophic lateral sclerosis.

## Proteomics biomarker discovery

**Figure 2:**
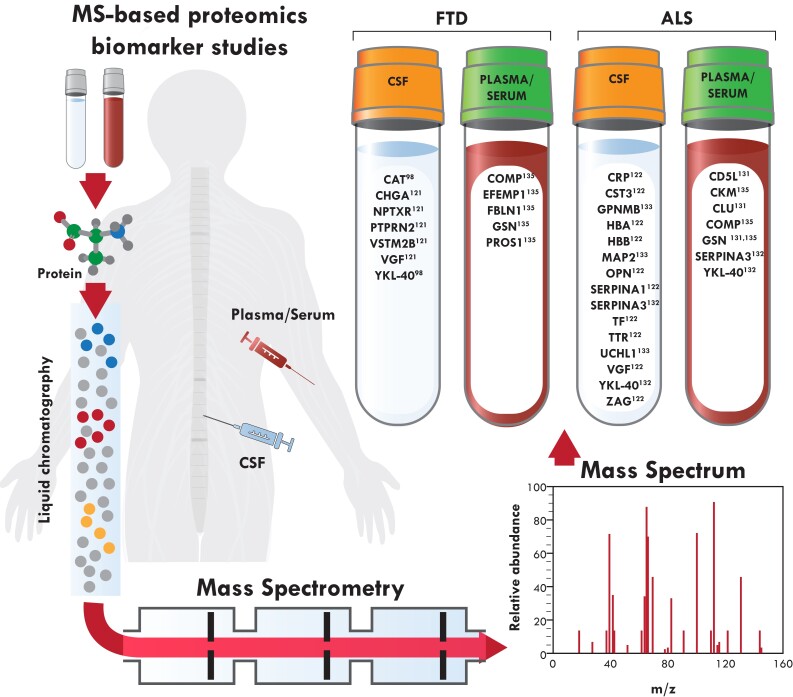
**Mass spectrometry-based proteomics biomarker development in frontotemporal dementia and amyotrophic lateral sclerosis.** Patient plasma/serum and CSF samples are processed and analysed by liquid chromatography and mass spectrometry. Proteins identified as potential biomarkers for frontotemporal dementia and amyotrophic lateral sclerosis are listed with their references.

Proteomics allows for unbiased global quantification of alterations in protein levels. Mass spectrometry (MS) is the current gold standard for proteomics analysis.^129^ MS proteomics is increasingly used in neurodegenerative diseases, particularly for biomarker development in Alzheimer’s disease^130^ and Parkinson’s disease.^131^ However, MS proteomics for analysis of frontotemporal dementia and amyotrophic lateral sclerosis biofluids has been hindered by a lack of validation across studies^100,132–134^ and so far MS proteomics in amyotrophic lateral sclerosis is more comprehensive than in frontotemporal dementia (Fig. 2).

In amyotrophic lateral sclerosis, one of the earliest studies identified CST3 and transthyretin (TTR) as potential biomarkers.^113^ A meta-analysis of this study and 10 subsequent studies in amyotrophic lateral sclerosis spanning a 10-year period (2005–16) identified 10 proteins (c-reactive protein, CST3, α-globin, β-globin, osteopontin, serpin A1, transferrin, TTR, nerve-growth factor and zinc-alpha-2-glycoprotein) as potential biomarkers in at least two independent studies^135^ (Fig. 2). For instance, CST3 was identified to be decreased in five studies.^113,136–139^ However, there were also some inconsistencies in the results across these studies. serpin A1, which was identified in three studies, was increased in amyotrophic lateral sclerosis in two studies^137,140^ but decreased in the third.^141^ Additionally, TTR was reported as significantly decreased in amyotrophic lateral sclerosis in three studies^113,142^ but increased in a fourth study.^143^ The publications included in this meta-analysis used a variety of different MS technologies, which may explain the inconsistency in the results. However, none of the 10 proteins identified for amyotrophic lateral sclerosis in the meta-analysis were validated in the four MS proteomics studies performed since.^134,144–146^ Interestingly, TTR and nerve-growth factor ‘inducible’ have also been identified in a subsequent publication as potential biomarkers for frontotemporal dementia.^133^

So far, only two studies have analysed frontotemporal dementia and amyotrophic lateral sclerosis biofluids side-by-side using MS. The first study used a combination of iTRAQ-based MS, multiple reaction monitoring and single-molecule array to identify and validate eight proteins (chitinase-3-like protein 2, crystallin alpha B, profilin-1, neural proliferation differentiation and control 1, ubiquitin carboxyl-terminal hydrolase L1, neuronal pentraxin receptor, triggering receptor expressed on myeloid cells 2 and transferrin receptor 1) with altered CSF levels in multiple *C9orf72* gene expansion cohorts and asymptomatic carriers.^147^ A more recent study, examining serum from frontotemporal dementia and amyotrophic lateral sclerosis patients identified 23 proteins altered in behavioural-variant frontotemporal dementia and 14 in amyotrophic lateral sclerosis serum. Of these identified proteins, 11 were altered in both diseases and six proteins were validated; cartilage oligomeric matrix protein, EGF containing fibulin extracellular matrix protein-1, FBLN1, gelsolin and protein S in frontotemporal dementia and creatinine kinase M-type, cartilage oligomeric matrix protein and gelsolin in amyotrophic lateral sclerosis.^148^ Inconsistency in the findings so far highlight the need for more thorough MS proteomics studies, particularly using plasma or serum. A possible explanation for the current inconsistency in results and lack of reproducibility may relate to different MS methodology and batch effects.

Other ‘omics’ approaches are also being used to screen for potential biomarkers and these include lipidomics in frontotemporal dementia^149^ and amyotrophic lateral sclerosis,^150,151^ metabolomics in frontotemporal dementia^152,153^ and amyotrophic lateral sclerosis.^154^ Most of the research are currently in an exploratory phase and it remains to be seen whether omics approaches can result in identification of biomarkers that are specific to frontotemporal dementia and amyotrophic lateral sclerosis. It is likely that, rather than a single biomarker, a panel of biomarkers that includes a combination of proteins, lipids and metabolites, is needed as an effective biomarker strategy for frontotemporal dementia and amyotrophic lateral sclerosis.

## Conclusion

Frontotemporal dementia and amyotrophic lateral sclerosis exist on a disease spectrum with overlapping biochemical and genetic traits. Currently, there are no specific fluid biomarkers for either disease. A number of proteins has been explored as potential fluid biomarkers for frontotemporal dementia and amyotrophic lateral sclerosis, particularly those relating to frontotemporal dementia/amyotrophic lateral sclerosis brain pathology, e.g. TDP-43, tau, C9ORF72 or SOD1. Other proteins, including NfL and immune-related markers YKL-40, GFAP and CST3 have also been explored as potential biomarkers, however, these markers are non-specific indicators of neurodegeneration or astrogliosis across the spectrum of neurodegeneration, thus limiting their utility specifically in frontotemporal dementia and amyotrophic lateral sclerosis.

Earlier research focused heavily on CSF, however, given the difficulty and invasiveness of collecting CSF from patients, there is an increased need for the development of blood-based biomarkers. Recent advances in MS have allowed for a greater detection and measurement of low abundant yet significantly altered proteins in serum and plasma. Consequently, mass spectrometry-based proteomics has come to the forefront as the method of choice for blood-based biomarker discovery.

So far, approaches to overcoming the various hurdles in biomarker development for frontotemporal dementia and amyotrophic lateral sclerosis, including a lack of specificity of biomarkers for frontotemporal dementia and amyotrophic lateral sclerosis given the expression of such markers in other neurodegenerative diseases, matrix issues when testing blood representative of what is expressed in CSF and inconsistencies in replicating results from different research groups, have been limited. Going forward, harnessing the current knowledge of the immune response, including its duration and whether it is a primary or secondary response to neurodegenerative processes, is crucial to identify sensitive and specific biomarkers of disease progression, particularly biofluid biomarkers that are non-invasive, accessible and already established for use in a clinical setting (i.e. blood and urine). An important strategy in the biomarker development will be the integration of genetics and multi-level omics approaches, which may be achieved by harnessing bioinformatics and deep-learning methods that may further integrate imputation of multi-omics and genetic datasets. An advantageous approach would be to collectively and comprehensively analyse all potential biomarkers that have been identified so far across frontotemporal dementia and amyotrophic lateral sclerosis using machine learning and AI technologies to identify common and/or overlapping proteins or pathways or molecular hits that can then be confirmed as informed targets to streamline biomarker discovery.

## Abbreviations

CST: cystatin
MS: mass spectrometry
NfH: neurofilament heavy
NfL: neurofilament light
NfM: neurofilament medium
p75^ECD^: urinary neurotrophin receptor p75 extracellular domain
TDP-43: TAR DNA binding protein 43
YKL-40: chitinase-3-like protein
